# Residual Viremia in Treated HIV^+^ Individuals

**DOI:** 10.1371/journal.pcbi.1004677

**Published:** 2016-01-06

**Authors:** Jessica M. Conway, Alan S. Perelson

**Affiliations:** 1 Department of Mathematics and Center for Infectious Disease Dynamics (CIDD), The Pennsylvania State University, University Park, Pennsylvania, United States of America; 2 Theoretical Biology and Biophysics, Los Alamos National Laboratory, Los Alamos, New Mexico, United States of America; Emory University, UNITED STATES

## Abstract

Antiretroviral therapy (ART) effectively controls HIV infection, suppressing HIV viral loads. However, some residual virus remains, below the level of detection, in HIV-infected patients on ART. The source of this viremia is an area of debate: does it derive primarily from activation of infected cells in the latent reservoir, or from ongoing viral replication? Observations seem to be contradictory: there is evidence of short term evolution, implying that there must be ongoing viral replication, and viral strains should thus evolve. However, phylogenetic analyses, and rare emergent drug resistance, suggest no long-term viral evolution, implying that virus derived from activated latent cells must dominate. We use simple deterministic and stochastic models to gain insight into residual viremia dynamics in HIV-infected patients. Our modeling relies on two underlying assumptions for patients on suppressive ART: that latent cell activation drives viral dynamics and that the reproductive ratio of treated infection is less than 1. Nonetheless, the contribution of viral replication to residual viremia in patients on ART may be non-negligible. However, even if the portion of viremia attributable to viral replication is significant, our model predicts (1) that latent reservoir re-seeding remains negligible, and (2) some short-term viral evolution is permitted, but long-term evolution can still be limited: stochastic analysis of our model shows that *de novo* emergence of drug resistance is rare. Thus, our simple models reconcile the seemingly contradictory observations on residual viremia and, with relatively few parameters, recapitulates HIV viral dynamics observed in patients on suppressive therapy.

## Author Summary

In HIV^+^ individuals, antiretroviral therapy (ART) effectively controls HIV viral loads to below levels detectable by routine tests. However, more sensitive tests can detect some residual viremia. The source of this virus is a matter of debate: does it derive from ongoing viral replication, or from viral production following activation of latently infected cells? Experimental observations support both sides of the argument: in patients on therapy, HIV shows no long-term evolution, and emergence of drug-resistant mutants is rare, implying no ongoing viral replication, but there remains short-term evolution, implying the opposite. To reconcile these observations, we propose a mathematical model of latently and productively infected cells and virus. Using our models we predict that, in patients on suppressive ART, the contribution of viral replication to residual virus, while small, yields short term-evolution. But even if the contribution is large, for example if adherence to therapy is poor, long-term evolution can still be limited, and *de novo* emergence of drug resistance is rare. Thus, our simple models reconcile the seemingly contradictory observations on residual viremia.

## Introduction

Antiretroviral therapy (ART) effectively controls HIV infection, suppressing HIV viral loads to below detectable levels in most patients. However, infection remains: cessation of treatment is usually followed by HIV rebound to high levels [1]. Ultra-sensitive assays, with detection thresholds as low as 0.3 virions per mL of plasma, reveal the presence of viremia in patients on treatment [2]. What is unclear is the source of this persistent, low-level viremia; does it derive from ongoing rounds of viral replication, or activation of infected cells in the latent reservoir, or some combination of the two [3]. Our aim is to employ simple mathematical models to gain insight into the source of residual viremia in HIV-infected patients.

HIV cell infection is usually followed by virus production and cell death. However, a small fraction of infected cells instead enter a state of latent infection [4, 5], in which HIV has integrated into the host cell DNA but there is little, if any, virus production. The virus’ cytopathic effects seem negligible, and these cells seem unaffected by therapy or host immune responses. The reservoir of these cells is established early during primary infection [6–8]. While in a latent state infected cells may undergo homeostatic proliferation [9], which promotes reservoir stability. The latent reservoir represents only a very small fraction of the total CD4^+^ T cell population but it is very long-lived; patients on treatment show a decaying reservoir with a half-life estimated to be between 6 and 44 months on average, so the time to complete eradication may be up to 70 years [10]. Eradication of the latent reservoir is considered to be one of the major hurdles to curing HIV infection [11]. Importantly, for our purposes, upon latent cell activation, viral production and ensuing cell death resume [12]. Mechanisms for the generation and maintenance of latency and subsequent activation remain unclear [4, 13, 14].

The evidence supporting latent cell activation as the only source of residual plasma viremia is as follows: (1) Intensification of ART, by adding an additional drug, has no appreciable impact on CD4 counts [15] or viral load [16]. (2) During suppressive ART, plasma virus shows little or no development of drug resistance mutations [17, 18]. (3) Clonal sequences of plasma virus indicate a close relationship with virus archived in the latent reservoir [19, 20]. (4) HIV envelope proteins in gut-associated lymphatic tissue show no evidence of evolution in patients on ART initiated during primary infection [21]. (5) Genotypic studies of pre- and post-treatment virus show a too-close relationship for the source of rebound virus to be ongoing viral replication [22].

However, there is also evidence supporting the notion of ongoing replication. For example, a genotypic study of episomal HIV cDNA collected prior to viral rebound showed evidence of recent evolution [23], suggesting that fresh rounds of cell infection with HIV contribute to residual viremia. Also, while the level of residual plasma viremia has been shown to correlate with the size of the CD4^+^ T cell viral reservoir in patients on ART, it does not correlate with markers of immune activation, suggesting that reactivation of the latent viral reservoir may not be the sole source of residual plasma viremia [24]. Residual viral replication may also occur in productively infected CD4^+^ T cells in various lymphoid tissues, without being reflected in plasma viremia [24, 25].

The mathematical modeling work below reconciles these contradictory observations. We make two underlying assumptions: that latent cell activation does occur in patients, and that *R*, the reproductive ratio, i.e., the average number of new cell infections induced by a single infected cell, during suppressive ART is less than 1. We show that, even though *R* < 1, the contribution of viral replication to residual viremia can be non-negligible if therapy is not sufficiently potent. Further, we shall show that, although the contribution of viral replication to residual viremia can be significant in such cases, low genetic variability can still be maintained, consistent with *de novo* emergence of drug resistance being very rare. Thus, recent evolution is possible, matching the observation in [23], but long term evolution is unlikely, matching the observations in [15, 17, 19, 20].

## Models

We begin with a standard model of viral dynamics with latent cell activation. Latently infected cells, *L*, proliferate at rate *ρ*, die at rate *μ*, and activate at rate *a* converting them into productively infected cells, *I*. Productively infected cells, *I*, die at rate *δ* and produce virions, *V*, at rate *p* per cell. Virions, *V*, are cleared at rate *c* per virion, and infect target cells, *T*, with mass-action infectivity rate constant *β* to make new infected cells, a fraction *f* of which become latently infected. We assume patients are on treatment such that the infectivity *β* is reduced by the factor (1 − *ε*), where *ε* is the effectiveness of therapy, 0 ≤ *ε* ≤ 1, with *ε* = 1 corresponding to 100% effective therapy. Formally reducing *β* by the factor (1 − *ε*) is appropriate for therapies involving reverse transcriptase inhibitors. However, one can show that, after a brief transient, it is also appropriate for protease inhibitors or a combination of the two types of drugs [26], which is the currently recommended therapy for HIV [27]. Observations of patients on long-term effective treatment show that the number of target cells *T* remains approximately constant; we therefore assume that *T* is constant. Taken together, we say that virions *V* penetrate cells at rate *βT* per virion, with a fraction (1 − *ε*) resulting in successful cell infection. Aborted cell infection still results in loss of the virion. For that reason, Eq (1) shows new cell infections, in the $\dot{L}$ and $\dot{I}$ equations, at rate (1 − *ε*)*βT*, but associated virion loss at rate *βT*.

**Fig 1:**
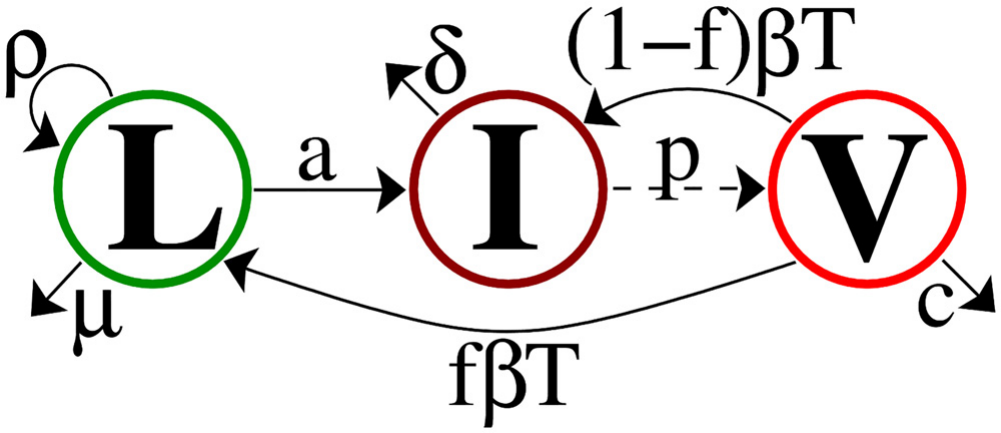
Model schematic.

The ordinary differential equations for the model shown in Fig 1 are

$$\begin{array}{ccc} \frac{dL}{dt} & = & f(1-\varepsilon )\beta TV+(\rho -a-\mu )L\\ \frac{dI}{dt} & = & aL+(1-\varepsilon )(1-f)\beta TV-\delta I\\ \frac{dV}{dt} & = & pI-(c+\beta T)V. \end{array}$$ (1)

To simplify this model we make the quasi-steady assumption *I* = (*c* + *βT*)*V*/*p*, since viral dynamics are known to be more rapid than infected cell dynamics [28, 29]. The system Eq (1) reduces to

$$\begin{array}{ccc} \frac{dL}{dt} & = & f\frac{(1-\varepsilon )\beta Tp}{c+\beta T}I+\left(\rho -a-\mu \right)L\\ \frac{dI}{dt} & = & aL+\left(1-f\right)\frac{(1-\varepsilon )\beta Tp}{c+\beta T}I-\delta I. \end{array}$$

Note that typically one sets *V* = *pI*/(*c* + *βT*) to obtain an equation for viral load *V*, with the aim of comparing model predictions with HIV viral load data [28, 29].

The on-treatment reproductive ratio *R*—the average number of infected cells in the second generation, starting from a single infected cell—is given, in the absence of latent reservoir dynamics, by

$$R=\frac{(1-\varepsilon )\beta Tp}{(c+\beta T)\delta }$$ (2)

(see S1 Text), which allows us to re-write the model equations more simply as

$$\begin{array}{ccc} \frac{dL}{dt} & = & fR\delta I+(\rho -a-\mu )L\\ \frac{dI}{dt} & = & aL-\delta \left[1-(1-f)R\right]I. \end{array}$$

Accounting for latent reservoir dynamics, the average number of new productive cell infections starting with a single productively infected cell, becomes

$$\begin{array}{c} R_{L}=R\left(1-f\left(1-\frac{a}{a+\mu -\rho }\right)\right) \end{array}$$ (3)

(for derivation, see S1 Text). This quantity is not the reproductive ratio for the model in Eq (1), which has a more complicated interpretation, since both *L* and *I* can be considered to be infected cell types. Since—as we will see below—*f*, the fraction of infections that lead to latency, is much smaller than 1, we neglect latent reservoir dynamics in the average given by Eq (3), and use the parameter *R*. Note that, for effective therapy, *ε* needs to be near enough to 1 that *R* < 1. Finally, for convenience, let −*η*_1_ = (*ρ* − *a* − *μ*) < 0, where *η*_1_ is the decay rate of the latent reservoir in absence of replenishment, i.e., the latent reservoir decay rate if the fraction of new cell infections resulting in latency, *f*, were zero, to obtain

$$\begin{array}{ccc} \frac{dL}{dt} & = & -\eta_{1}L+fR\delta I\\ \frac{dI}{dt} & = & aL-\delta \left[1-(1-f)R\right]I. \end{array}$$ (4)

We will employ variants of this model to investigate viral dynamics in the presence of suppressive therapy.

### Key parameters: Reproductive ratio *R* and latent fraction *f*

The reproductive ratio *R* = (1 − *ε*)*pβT*/*δ*(*c* + *βT*) is a key parameter in our model in determining the amount of residual replication. The fraction *f* determines the level of predicted latent reservoir re-seeding in patients on treatment, which can be significant if *R* is large. These parameters are therefore central in characterizing ongoing viral dynamics in patients on treatment. We now discuss realistic ranges for those parameters.

#### Reproductive ratio *R*

In patients on effective treatment, the reproductive ratio *R* = (1 − *ε*)*pβT*/(*c* + *βT*)*δ* must be less than 1, since treatment controls infection. Table 1 summarizes various parameter estimates in the literature and gives *c* = 23–100 day^−1^, *p* = 1,000–50,000 day^−1^, *T* = 800 cells per μL (typical for a patient on long-term suppressive ART), and *δ* = 1 day^−1^. Taken together these imply that *R* will range between (1 − *ε*) (8 × 10^6^ *β* − 6.4 × 10^7^ *β* ^2^ + …) ≈ 8 × 10^6^(1 − *ε*)*β* and (1 − *ε*) (1.7 × 10^9^ *β* − 6.0 × 10^13^ *β* ^2^ + …) ≈ 1.7 × 10^9^(1 − *ε*)*β*, with the mass-action infectivity rate, *β*, remaining unclear. Nonetheless we used the Taylor series of *R* in terms of *β* since we anticipate *β* to be small [28, 30, 31]. *ε* is left as a parameter since we will later explore drug efficacy.

**Table 1:** Initial conditions and baseline parameters, with parameter ranges given in parentheses.

Parameter	Meaning	Value	Source
*c*	viral clearance rate	23 day^−1^ (23 day^−1^–100 day^−1^)	lower: [35] upper: [36]
*δ*	productively infected cell death rate	1 day^−1^	[37]
*p*	viral production rate	50000 per day (1000–50 000 per day)	lower: [38], upper: [39]
*ε*	drug efficacy	0.99 (0.9–0.999)	See text
*T*	target cell density (assumed constant)	800 cells per μL	
$t_{1/2}^{L}$	Net latent reservoir half-life	44 months	[10]
*η*_1_	Latent reservoir decay rate in the absence of replenishment by *de novo* infection	5.17 × 10^−4^ day^−1^; half-life is ln(2)/*η*_1_ = 44.0 mos	See text
*L*_0_	Initial latent reservoir size	1 per 10^6^ cells ≈ 0.8 cells per mL	[40]
*V*_0_	Initial viral load	3.1 copies/mL	[2]
*a*	Latent cell activation rate	1.74 × 10^−3^ day^−1^	See text

Instead of using estimates for *β*, take *R*_*u*_, the reproduction number in an untreated individual, i.e. *ε* = 0, to be *R*_*u*_ ≈ 1. In the absence of therapy patients attain an approximately constant set-point viral load. At the set-point, *R*_*u*_ must equal 1 or else viral load would increase (*R*_*u*_ > 1) or decrease (*R*_*u*_ < 1). With *R*_*u*_ ≈ 1, *β* ≈ 5.75 × 10^−10^ − 1.25 × 10^−7^ mL^−1^day^−1^, in the range of existing estimates for *β* [30, 32]. Further, *R*/*R*_*u*_ ≈ (1 − *ε*)*T*/*T*_*u*_ since *βT* ≪ *c*. Assuming target cell density in the absence of therapy *T*_*u*_ = 350 cells per μL, below which therapy is recommended [27], and an on-treatment target cell density of *T* = 800 cells per μL (see Table 1), *R* ≈ 2.3(1 − *ε*). In the following we define R* = 2.3 as the reproductive ratio if treatment is halted. Note that for acute HIV infection, the basic reproductive number was estimated to be 2.77 [33], after adjusting the estimate in [33], since in our model we neglect the lag between cell infection and viral production.

Then if we assume a drug efficacy in the range *ε* = 0.9–0.999, the reproductive ratio *R*, in the presence of therapy, is in the range 2.3 × 10^−3^ − 2.3 × 10^−1^.

#### Latent cell fraction *f*

Archin et al. (2012) used a mathematical model to investigate the seeding of the latent reservoir. They derived model predictions for the frequency of CD4^+^ cells that are latently infected, and showed that their predictions correlate well with measurements of latent cell infections, shown in Fig. D in S1 Text.

The correlation derived in [34] showed that the log of their model prediction, given in units of of 10^14^ *fβ*, has a linear relationship with the log of the measured latent cell frequency. Specifically they showed that log_10_ *L*_*p*_ = 0.35log_10_ *L*_*m*_ − 0.35, where *L*_*p*_ and *L*_*m*_ indicate the predicted and measured latent reservoir size per 10^6^ CD4^+^ cells, respectively, with *L*_*p*_ in arbitrary units 10^14^ *fβ*, as shown in Fig. D in S1 Text.

Typically, in chronically infected patients, the measured latent reservoir size is on the order of *L*_*m*_ = 1 per 10^6^ CD4^+^ cells [4]. From the relationship log_10_ *L*_*p*_ = 0.35log_10_ *L*_*m*_ − 0.35, Archin et al. (2012)’s corresponding modeling prediction would give *L*_*p*_ = 0.1 × 10^14^ *fβ* per 10^6^ CD4^+^ cells. Comparing the measurement *L*_*m*_ and prediction *L*_*p*_ directly,

$$1\;\text{per}\;10^{6}\;\text{cells}=0.1\times 10^{14}f\beta \;\text{per}\;10^{6}\;\text{cells},$$

since the prediction is in arbitrary units of 10^14^ *fβ*. In the previous section we estimated the infectivity *β* ≈ 1.3 × 10^−9^ − 2.9 × 10^−7^ mL^−1^day^−1^. Thus we find that *f* ≈ 3.4 × 10^−7^ − 7.7 × 10^−5^, which is very small compared to 1. We use the upper bound *f* = 10^−4^ as our baseline fraction of new infections that lead to latency.

### Remaining model parameters

Our primary results below rely upon the reproductive ratio *R* = (1 − *ε*)*pβT*/(*c* + *βT*)*δ* only, since *f* is small. However, for the purposes of illustrative simulation, we input the parameters individually rather than as the group parameter *R*. Where possible, model parameter estimates are taken from the literature [2, 10, 35–40], as listed in Table 1.

For most antiretroviral therapy, the associated *in vivo* drug efficacy *ε* is poorly characterized. Recently raltegravir, an integrase inhibitor, has been estimated to have efficacy 0.94 in a combination therapy including emtricitabine and tenofovir disoproxil fumarate, and 0.997 during monotherapy [41]. Integrase inhibitors are not yet included in most recommended antiretroviral therapy combinations [27], but combination therapy with raltegravir seems to be no more effective than other types of drugs in treatment-naïve patients [42, 43]. We therefore choose for our baseline net drug efficacy *ε* = 0.99, slightly better than the efficacy of raltegravir in the combination therapy used by Andrade et al. (2015) [41].

We will use this drug efficacy to fix the latently infected cell activation rate, next, assuming the viral load on long-term therapy *V*_0_ = 3.1 copies/mL [2]. With *a* fixed at this value we will then explore the sensitivity of our results to drug efficacies in the range *ε* = 0.9–0.999.

Beyond the net measured latent reservoir half-life, $t_{1/2}^{L}=\ln\left(2\right)/\eta_{2}$, model parameters relating to latent reservoir dynamics, *η*_1_ and *a*, remain poorly understood. However, the largest negative eigenvalue in our model (4) should correspond to the observed long term decay of latently infected cells, *η*_2_. We choose the latent reservoir decay rate in the absence of replenishment by *de novo* infection, *η*_1_, as a function of this net latent reservoir decay *η*_2_. As shown in Sec. B.3 in S1 Text,

$$\eta_{1}=\eta_{2}-\frac{a\delta fR}{\eta_{2}-\delta \left(1-\left(1-f\right)R\right)}.$$ (5)

We choose the latent cell activation rate *a* so that in model (4), at some arbitrary time after being on therapy for a long period, designated *t* = 0, the latent reservoir size *L*_0_ and viral load *V*_0_ are in quasi-equilibrium, i.e.,

$$a=\left(\delta \left(1-\left(1-f\right)R\right)-\eta_{2}\right)\frac{cV_{0}}{pL_{0}},$$ (6)

see Sec. B.3 in S1 Text for details. Note that this approach imposes an additional constraint on our parameters; *a* > 0 requires that *δ* [1 − (1 − *f*)*R*] > *η*_2_. We interpret this constraint as the net decay rate of productively infected cells in the presence of new infections but in the absence of new latent cell activations, *δ* [1 − (1 − *f*)*R*] (c.f. Eq (4)) must be more rapid than the net decay rate of the latent reservoir, *η*_2_. Assuming a drug efficacy of *ε* = 0.99, an on-therapy quasi-steady state viral load *V*_0_ = 3.1 copies/mL, and corresponding latent reservoir size *L*_0_ = 1 per 10^6^ cells, we obtain a baseline activation rate of *a* = 1.74 × 10^−3^ *day* ^−1^, which corresponds to an average time of activation for a single latently infected cell of 575 days. This is about 3.5 times the estimated lifespan of a human memory CD4^+^ T-cell [44], so only a minority of latently infected cells are expected to become activated before they die. Nonetheless, we estimate that there are *aL* ≈ 174 latent cell activations per day, assuming 10^11^ CD4^+^ T-cells body-wide. Pinkevych et al. (2015) estimated that on average, after therapy is interrupted, active viral replication is initiated once every 6 days. This does not imply that there is an average of one new latent cell activation every six days, as there also needs to be ensuing rounds of viral replication following the activation of a latently infected cell that cause viral rebound, rather than a chain of infection that ultimately dies out. Therefore, the actual value of *aL* remains unclear. We use *aL* ≈ 174 latent cell activations per day but our qualitative results are not sensitive to this choice, see Sec. E in S1 Text.

When investigating viral dynamics under drug efficacies *ε* ≠ 0.99 we recalculate the associated reproductive ratio, *R* = (1 − *ε*)R* and then re-compute the associated on-therapy quasi-steady state viral load, *V*_0_ = *apL*_0_/*c*(*δ*(1 − (1 − *f*)*R*) − *η*_2_) from Eq (6).

## Results

### On the contribution of ongoing viral replication to residual plasma viral load

#### Model extension: Two productively infected cell compartments

In order to determine the extent of new cell infections in a patient under therapy, we split the productively infected cell compartment, *I*, into two parts: *I*_*a*_, infected cells generated by latent cell activation, and *I*_*r*_, infected cells generated by viral replication. The model (4) is extended to become

$$\begin{array}{ccc} \frac{dL}{dt} & = & -\eta_{1}L+fR\delta \left(I_{a}+I_{r}\right)\\ \frac{dI_{a}}{dt} & = & aL-\delta I_{a}\\ \frac{dI_{r}}{dt} & = & \left(1-f\right)\delta R\left(I_{a}+I_{r}\right)-\delta I_{r} \end{array}$$ (7)

We are interested in exploring viral dynamics in patients on long-term treatment, with time *t* = 0 representing an arbitrary time after the initiation of treatment and after transient viral load dynamics have passed.

The eigenvalues of the system Eq (7) are

$$\begin{array}{ccc} \lambda_{1} & = & -\delta \\ \lambda_{\pm } & = & \frac{1}{2}\left(-\eta_{1}-\delta \left(1-(1-f)R\right)\pm \sqrt{(\eta_{1}-\delta {\left(1-(1-f)R)\right)}^{2}+4a\delta fR}\right), \end{array}$$ (8)

with associated eigenvectors

$$\begin{array}{ll} {\vec{v}}_{1} & ={\left(\begin{array}{ccc} 0, & -1, & 1, \end{array}\right)}^{T}\\ {\vec{v}}_{\pm } & =\left(\begin{array}{c} 2a\delta fR+(\delta -\eta_{1})\left\{-\eta_{1}+\delta \left[1-(1-f)R\right]\pm \sqrt{{\left(\eta_{1}-\delta (1-(1-f)R)\right)}^{2}+4a\delta fR}\right\}\\ a\left\{-\eta_{1}+\delta \left[1-(1-f)R\right]\pm \sqrt{{\left(\eta_{1}-\delta (1-(1-f)R)\right)}^{2}+4a\delta fR}\right\}\\ 2a\delta (1-f)R \end{array}\right). \end{array}$$

Since all parameters are positive, and 0 < *f* < 1 and 0 < *R* < 1, one can show that both eigenvalues *λ*_±_ are negative (see S1 Text). The larger eigenvalue *λ*_+_ is closer to zero than either *λ*_−_ or *λ*_1_ = −*δ*, and ${\vec{v}}_{+}$ is therefore the slow manifold of the fixed point (*L*, *I*_*a*_, *I*_*r*_) = (0, 0, 0) [45]. After some period on long term therapy, the subsequent dynamics correspond to dynamics along the slow manifold described by the eigenvalue *λ*_+_ and corresponding eigenvector ${\vec{v}}_{+}$. That is, over long times, the latent and productive cell numbers decay at rate |*λ*_+_|, with relative magnitudes corresponding to their respective eigenvector ${\vec{v}}_{+}$ values. The decay in the latently and productively infected cell numbers at rates |*λ*_1_| and |*λ*_−_| happen on faster time scales, since |*λ*_1_| > |*λ*_+_| and |*λ*_−_| > |*λ*_+_|, and therefore represent transient, short-term dynamics only. Our initial conditions should lie along the eigenvector ${\vec{v}}_{+}$; taking the initial number of latently infected cells to be *L*_0_, we set the initial *I*_*a*_(0) so that $L_{0}/I_{a}\left(0\right)={\vec{v}}_{+}^{\left(1\right)}/{\vec{v}}_{+}^{\left(2\right)}$, and the initial *I*_*r*_(0) so that $L_{0}/I_{r}\left(0\right)={\vec{v}}_{+}^{\left(1\right)}/{\vec{v}}_{+}^{\left(3\right)}$, where ${\vec{v}}_{+}={\left(\begin{array}{ccc} {\vec{v}}_{+}^{\left(1\right)} & {\vec{v}}_{+}^{\left(2\right)} & {\vec{v}}_{+}^{\left(3\right)} \end{array}\right)}^{T}$. Thus the initial conditions on *I*_*a*_(*t*) and *I*_*r*_(*t*) are

$$\begin{array}{ccc} I_{a}\left(0\right) & = & \frac{a\left\{-\eta_{1}+\delta \left[1-(1-f)R\right]+\sqrt{(\eta_{1}-\delta {\left(1-(1-f)R)\right)}^{2}+4a\delta fR}\right\}}{2a\delta fR+\left(\delta -\eta_{1}\right)\left\{-\eta_{1}+\delta \left[1-(1-f)R\right]+\sqrt{(\eta_{1}-\delta {\left(1-(1-f)R)\right)}^{2}+4a\delta fR}\right\}}L_{0},\;\text{and}\\ I_{r}\left(0\right) & = & \frac{2a\delta (1-f)R}{2a\delta fR+\left(\delta -\eta_{1}\right)\left\{-\eta_{1}+\delta \left[1-(1-f)R\right]+\sqrt{(\eta_{1}-\delta {\left(1-(1-f)R)\right)}^{2}+4a\delta fR}\right\}}L_{0}. \end{array}$$

More significantly, the fraction of infected cells that come from residual viral replication, in patients on long-term treatment, is

$$\frac{I_{r}}{I_{a}+I_{r}}=\frac{v_{+}^{\left(3\right)}}{v_{+}^{\left(2\right)}+v_{+}^{\left(3\right)}}=\frac{2\delta (1-f)R}{\delta \left(1-f\right)R-\eta_{1}+\delta +\sqrt{(\eta_{1}-\delta {\left(1-(1-f)R)\right)}^{2}+4a\delta fR}}.$$ (9)

We can also use the eigenvector to determine the critical reproductive ratio *R* = *R*_*c*_ where the contributions to the total number of newly infected cells, *I*, from latent cell activation, *I*_*a*_, and viral replication, *I*_*r*_, are equal. We set ${\vec{v}}_{+}\left(2\right)={\vec{v}}_{+}\left(3\right)$ and solve for *R* to obtain

$$R_{c}=\frac{\delta -\eta_{2}}{2\delta (1-f)}.$$ (10)

If *R* > *R*_*c*_, viral replication makes the dominant contribution to the number of new productively infected cells. If *R* < *R*_*c*_, the contribution of viral replication is small, and latent cell activation dominates. Recall that the reproductive ratio *R* is defined here as *R* = (1 − *ε*)*pβT*/(*c* + *βT*)*δ* and is independent of latent cell infection. Since the infected cell death rate *δ* is 1 *day* ^−1^ (see Table 1), while the latency fraction *f* and latent reservoir net decay rate *η*_2_ are small compared to 1, *R*_*c*_ ≈ 0.5.

#### Ongoing viral replication and plasma viral load

The critical reproductive ratio *R*_*c*_, Eq (10), for realistic, small values of *f*, is *R*_*c*_ ≈ 0.5. Since *R* is in the range 2.3 × 10^−3^ − 2.3 × 10^−1^, *R* < *R*_*c*_ and the dominant contribution to plasma viremia in patients on treatment comes from activation of latently infected cells.

However, the contribution to residual viremia from viral replication may be non-negligible. We can re-write the ratio *I*_*r*_/*I* as

$$\frac{I_{r}}{I_{a}+I_{r}}\approx \frac{(1-f)\delta }{\delta -\eta_{1}}R,$$ (11)

which is the first term in the Taylor series for *I*_*r*_/(*I*_*a*_ + *I*_*r*_) in Eq (9) about *R* = 0; since 0 < *R* < 1, higher order terms will become very small, and even the Taylor series coefficient of *R* ^2^, *af*(1 − *f*)*δ* ^2^/(*δ* − *η*_1_)^3^, is very small since *f* ∼ *O*(10^−6^) − *O*(10^−4^). Because the viral load is proportional to the number of infected cells, the fraction of circulating virus associated with viral replication is approximately given by Eq (11), which can be used with improved estimates for latent fraction *f* and reproductive ratio *R* to simply estimate the fraction of circulating virus associated with ongoing viral replication. Since *f* ∼ *O*(10^−6^) − *O*(10^−4^), and *δ* ≫ *η*_1_, we can approximate Eq (11) further to obtain

$$\frac{I_{r}}{I_{a}+I_{r}}\approx R.$$ (12)

Using this, we calculate that *I*_*r*_/*I* ≈ 2.3% with our baseline parameters (*R* = 0.023).

**Fig 2:**
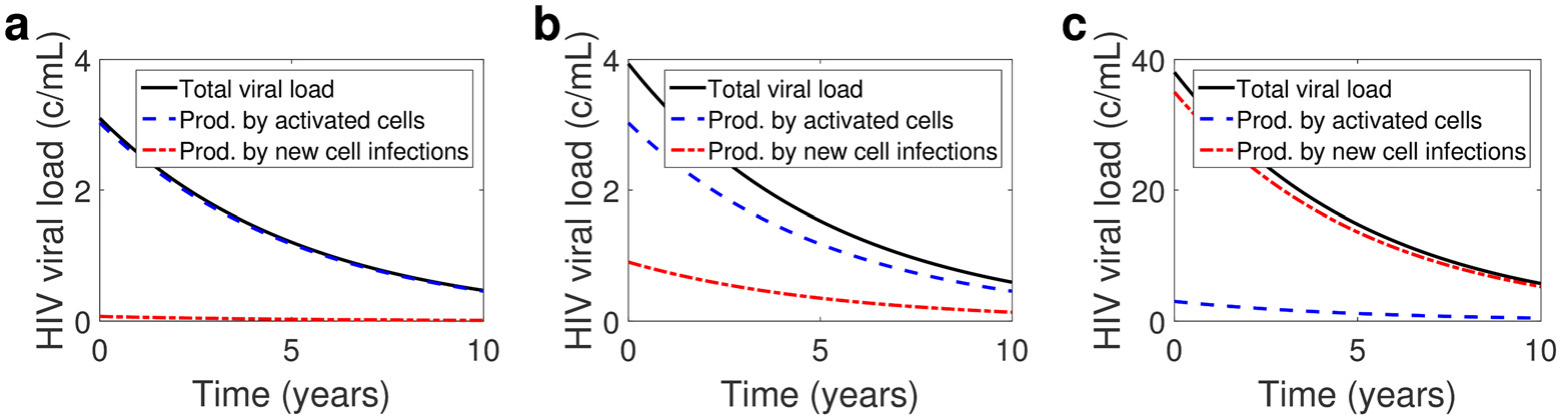
Total HIV RNA copies per mL (black, solid line), on a linear scale, with contributions from activated latently infected cells (blue, dashed line) and from newly infected cells (red, dash-dotted lines) for fraction of infections leading to latency *f* = 10^−4^ and reproductive ratio (a) *ε* = 0.99 (*R* = 0.023, *V*_0_ ≈ 3 copies/mL), (b) *ε* = 0.9 (*R* = 0.23, *V*_0_ ≈ 4 copies/mL), and (c) *ε* = 0.6 (*R* = 0.92, *V*_0_ ≈ 38 copies/mL). Other parameters set to *δ* = 1 day^−1^ and $t_{1/2}^{L}=44$ months.

Fig 2 shows on-treatment viral dynamics for different values of *R* and associated residual plasma viral load. We assume that

$$V_{0}\frac{=apL_{0}}{c\left(\delta \left(1-\left(1-f\right)\left(1-\varepsilon \right)R^{*}\right)-\eta_{2}\right)}=3.1\;\text{copies}/\text{mL},$$

from Eq (6), with R* = 2.3 and baseline drug efficacy *ε* = 0.99, to fix *a*. We recover similar results with *a* fixed using different baseline drug efficacies, *ε* = 0.6–0.999 (see Sec. E in S1 Text), since the relationship Eq (12) does not depend on *a*. Fig 2a and 2b show that the contributions of viral replication to the total viral load assuming drug efficacy *ε* = 0.99 and 0.9, are 2.3% and 23%, respectively, approximated well by *R* = 2.3(1 − *ε*). Our model predicts that in the case of *ε* = 0.999 (figure not shown), in the range of raltegravir monotherapy [41], the contribution of viral replication to the total viral load is negligible, approximately 0.23%.

Fig 2c shows viral dynamics assuming a low drug efficacy *ε* = 0.6, below our range of primary consideration, which gives *R* = 0.92. In that case approximately 92% of circulating virus would be associated with ongoing viral replication. However, we claim that a drug efficacy of *ε* = 0.6 is not realistic under current regimens. The drug efficacy *ε* = 0.6 is associated with a viral load of 38 copies/mL, a value below the limit of detection of conventional clinical assays. Under recent regimens, the median measured viral load was 3.1 copies/mL [2], and though it is reasonable to assume that drug efficacy has improved significantly since the roll out of ART, even in 1999 the mean viral load, in well-suppressed patients, was reported as 17 copies/mL [46]. Therefore, an average drug efficacy of *ε* = 0.6 is only realistic if the virus has developed some resistance to therapy or if patient adherence is low, in which cases we would expect more viral replication.

#### Ongoing viral replication and the potential for mutation

Ongoing viral replication in patients on effective therapy, however slight, carries with it the probability that the virus may mutate. We now consider the lineage created by newly activated, latently infected cells in our simple model.

**Fig 3:**
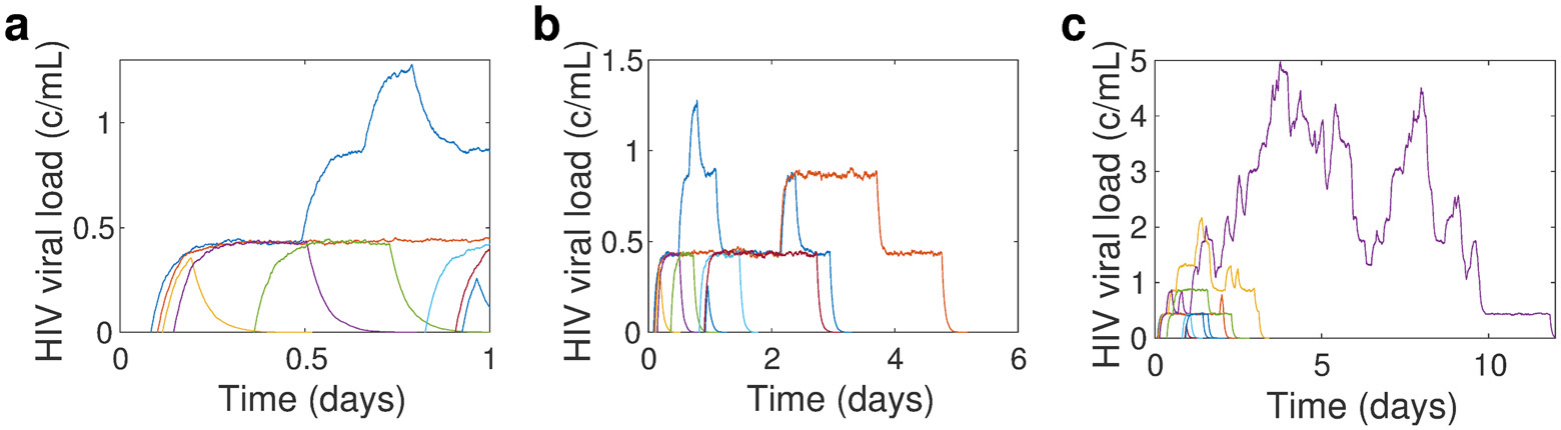
Stochastic simulation realization [47] of viral load dynamics resulting from a single day’s latent cell activations for (a-b) drug efficacy *ε* = 0.9, associated with *R* = 0.23 and mean viral load *V*_0_ ≈ 4 copies/mL, and (c) *ε* = 0.6, associated with *R* = 0.92 and mean viral load *V*_0_ ≈ 38 copies/mL. In (a) we show the time span of a single day, to illustrate a single day’s dynamics, while in (b-c) we show dynamics until the lineages created by all activations from a single day, die out. Note that in each case, the latent cell activations occur at the same time. The latent reservoir decay rate *η*_2_ = log(2)/44 months and *aL*_0_ is chosen so that the on-therapy quasi-steady state viral load is *V*_0_ = 3.1 copies/mL assuming baseline drug efficacy *ε* = 0.99, infected cell death rate *δ* = 1 day^−1^, and latency fraction *f* = 10^−4^.

Since the reproductive ratio *R* < 1, the lineage of infected cells generated from the activation of a single latently infected cell goes extinct as *t* → ∞. Fig 3 shows illustrative realizations of stochastic viral dynamics in the blood, following the stochastic activation of latently infected cells, over a single day, for *R* = 0.23. Each trajectory’s dynamics is given by the branching process analogue of the differential equations model assuming a constant number of target cells *T*,

$$\begin{array}{ccc} \dot{I} & = & \beta TV-\delta I\\ \dot{V} & = & pI-(c+\beta T)V, \end{array}$$

with initial condition *I*(0) = 1, since dynamics are initiated by the activation of a single latently infected cell, simulated using the stochastic simulation algorithm [47]. Assuming a blood volume of 5 liters, *aL* ≈ 7 day^−1^ is the Poisson rate parameter for the activation times of latently infected cells in the blood; Fig 3a shows 8 latently infected cell activations, each in a different color. The steps in viral load, shown best in the blue curve in Fig 3a, indicate new cell infections, hence an increase in the produced viral load. Since *R* < 1, the lineages created by the activation of latently infected cells ultimately go extinct, after some, but not many, rounds of viral replication, as shown in Fig 3b. How long the lineages endure, that is, how many rounds of viral replication occur before extinction, depends on *R*. Fig 3c shows a stochastic realization of viral dynamics following latent cell activations at the same times as in Fig 3a and 3b, but now with *R* = 0.92. Note the difference in timescales between Fig 3b and 3c: for *R* = 0.92, i.e., *ε* = 0.6, there are more rounds of viral replication, and the lineages created by the activation of a single latently infected cell last longer than in the *R* = 0.23 case. These illustrative stochastic simulations make clear why, given identical latent cell activation times, the contribution of ongoing viral replication depends on the drug effectiveness and hence, on *R*. The total viral load is the sum of the viral load curves shown in Fig 3a–3c, and since for larger *R*, there are more rounds of viral replication before extinction, the lineages contribute to viral load for a longer amount of time (see Fig. E in S1 Text).

When considering viral evolution, the number of generations, i.e. rounds of viral replication, before the lineage goes extinct becomes important. The number of generations is proportional to the number of “chances” for mutations to take place.

Here we briefly outline the derivation of the probability distribution of the number of rounds of viral replication before extinction, with the full derivation given in the S1 Text. Following [48], define *α*_*j*_ as the number of infected cells in generation *j*, i.e., the number of infected cells remaining after *j* rounds of viral replication, and let *G* denote the number of rounds before extinction, so that *G* = *k* when *α*_*k*_ ≥ 1 and *α*_*k*+1_ = 0. The cumulative distribution of the number of rounds of viral replication arising from a single infected cell is defined as *f*_*k*_ = *Pr*{*G* ≤ *k*|*α*_0_ = 1}. Then the probability of the lineage surviving for *k* rounds of replication, *f*_*k*_, depends on the probability of surviving to the (*k* − 1)^st^ round, *f*_*k*−1_, and on the number of infected offspring from the cells present at the (*k* − 1)^st^ round, giving the recurrence relation *f*_*k*_ = *h*(*f*_*k*−1_), *f*_−1_ = 0 [48]. *h*(*x*) is the probability generating function for the offspring distribution, i.e., the distribution of the number of infected cells produced from a single infected cell in one generation; in this case, *h*(*x*) = 1/(1 − *R*(1 − *x*)) (see S1 Text). Solving the recurrence relation we find *f*_*k*_ = (1 − *R* ^*k*+1^)/(1 − *R* ^*k*+2^).

Thus, the cumulative probability distribution for the number of rounds of viral replication following the activation of a latently infected cell is given by

$$f_{k}=\text{Cumulative}\;\text{prob}\;\text{of}\;k\;\text{or}\;\text{fewer}\;\text{rounds}\;\text{of}\;\text{replication}\;=\frac{1-R^{k+1}}{1-R^{k+2}}.$$ (13)

Then the probability of exactly *k* rounds of viral replication, given a single latent cell activation, is *g*_*k*_ = *f*_*k*_ − *f*_*k*−1_ (see S1 Text for derivation).

**Fig 4:**
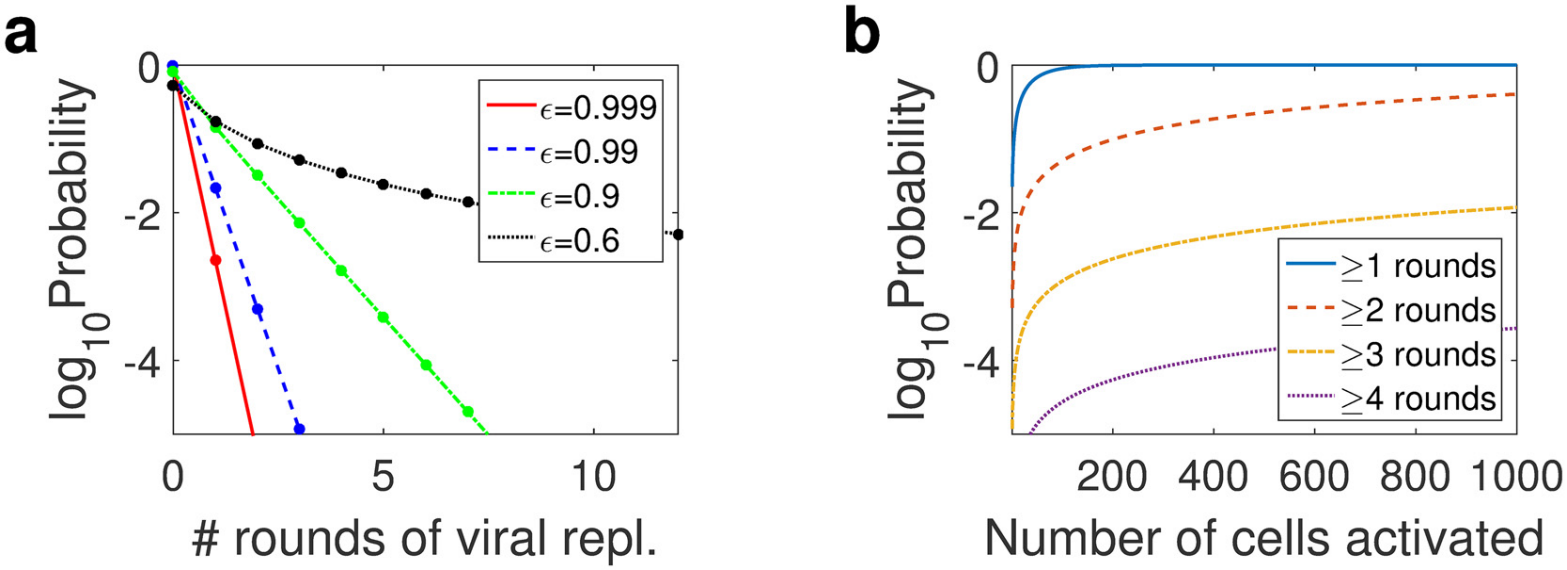
Probability distribution of number of viral replications following activation of a latently infected cell. (a) Probability of the number of rounds of viral replication following the activation of a single latently infected cell, assuming drug efficacy *ε* = 0.999, 0.99, and 0.9, which are associated with reproductive ratios *R* = 0.0023, 0.023, and 0.23, and initial viral loads *V*_0_ = 3, 3.1, and 4 copies/mL, respectively. (b) The probability of the maximum number of rounds of viral replication achieved as a function of the number of latent cell activations, using *ε* = 0.99, which is associated with *R* = 0.023 and initial viral load *V*_0_ = 3.1 copies/mL. Note that these are discrete distribution functions, with the dots in (a) indicating probability of cells achieving *k* generations; the lines are included for clarity and have no meaning.

Fig 4a shows the probability of *k* rounds of viral replication following the activation of a single latently infected cell for drug efficacies *ε* = 0.9, 0.99, and 0.999. A drug efficacy of *ε* = 0.9 yields more rounds of viral replication, with higher probability, than higher drug efficacies *ε* = 0.99 and 0.999. For *ε* = 0.9, *R* = 0.23 and viral replication contributes 23% of the total plasma viremia, while for *ε* = 0.999, *R* = 0.0023 and viral replication contributes 0.23% of the total plasma viremia. Since for each *ε* the influx of activated latently infected cells, *aL*, is assumed to be the same, the additional rounds of viral replication are the source of the larger contribution of viral replication to plasma viremia for smaller *ε*/larger *R*.

But even for our baseline *ε* = 0.99, we predict that there is a 2.3% chance that there will be at least one round of replication, see Table 2. This implies that, on average, out of 50 new latently infected cell activations, at least one will result in multiple rounds of viral replication. In the extreme case of a drug efficacy of *ε* = 0.6, realistic only if we assume compliance with therapy is low or that the virus has developed some resistance to therapy, multiple rounds of replication are far more likely. This explains the stochastic viral dynamics realization shown in Fig 3c. The probability of three or more rounds of viral replication is greater than 21% (see Table 2). But for *ε* = 0.6, the probability of exactly *k* rounds of replication decays more slowly than for *ε* = 0.9, 0.99, or 0.999, as shown in Fig 4a, and the probability of more than 50 rounds of viral replication is 12.55%, while the probability of more than 100 rounds is 0.002%, see Fig. Fc in S1 Text. More rounds of replication offer more opportunities for drug resistant mutants to arise, which matches intuition: drug resistant mutants arise more readily with lower drug efficacy.

**Table 2:** Probability of at least one, two, or three rounds of viral replication, following the activation of a single latently infected cell, depending on the drug efficacy *ε* = 0.999, 0.99, 0.9, and 0.6, which are associated with different reproductive ratios *R*, 0.0023, 0.023, 0.23, and 0.92, respectively, and give different initial viral loads *V*_0_, 3 copies/mL, 3.1 copies/mL, 4 copies/mL, and 38 copies/mL, respectively.

	*ε* = 0.999	*ε* = 0.99	*ε* = 0.9	*ε* = 0.6
≥1 round of viral replication	0.23%	2.25%	18.70%	47.92%
≥2 rounds of viral replication	0.0005%	.05%	4.12%	30.60%
≥3 rounds of viral replication	1 × 10^−6^%	0.001%	0.94%	21.97%

The stochastic realization shown in Fig 3 (restricted to the blood, averaging 7 activations/day, for illustrative purposes) shows that the viral lineages created by latent cell activations co-exist. As the frequency of latent cell activations, and therefore the number of co-existing lineages, increases, so does the probability that some circulating virus derives from productively infected cells arising from rounds of replication. Given *α*_0_ latent cell activations, the probability that at least one of those activations will result in *k* or more rounds of viral replication is $1-f_{k}^{\alpha_{0}}$. Fig 4b shows the probability of latently infected cells initiating ≥1, 2, 3, or 4 rounds of replication as a function of the number of cells. Note that these are increasing with the number of starting cells activated, and the probability of having cells with more than 1 round of replication goes to 100% relatively quickly, given 100 cell activations. The high probability of at least one round of viral replication given 100 activations is also true if we assume drug efficacies *ε* = 0.6, *ε* = 0.9 and *ε* = 0.999 (see Fig. F in S1 Text for these cases). This model prediction is in line with clinical observations of fresh rounds of viral replication in patients on therapy [23].

The number of new cell activations per day is given, in our model, by *aL*, the product of the activation rate and the latent reservoir size. At our arbitrary point on long-term therapy after a new, low, viral load set-point has been reached, i.e., *t* = 0,

$$aL_{0}=\left(\delta \left(1-\left(1-f\right)R\right)-\eta_{2}\right)\frac{cV_{0}}{p}.$$

For our baseline parameters (see Table 1) including an initial viral load *V*_0_ = 3.1 copies/mL and *R* = 0.023, this corresponds to approximately 174 activations per day, neglecting latent reservoir decay, which is very slow. Extending *R* to the range 0.0023–0.23 gives approximately 137–178 activations per day. These model predictions however are very sensitive to parameter choices; for example, for a smaller viral production rate *p* = 2000 copies/day, in line with estimates from [38], *R* in the range 0.0023–0.23 gives approximately 3400–4400 new latent cell activations per day. However, as discussed in **Remaining model parameters**, these predictions far exceed the lower bound of one activation every six days [49]. We note further that we model dynamics of activation very simply; importantly for this discussion, we assume no clonal expansion, which—since latently infected cells are mostly memory cells [9]—could follow if activation is triggered by antigenic stimulation [50, 51]. Thus a single activation could rapidly yield more infected cells, which, in turn, would increase the probability that cells surviving multiple rounds of viral replication are circulating in a patient on suppressive therapy (cf. Fig 4b). However, regardless of drug efficacy, and other than an initial jump in probability with the first few latent cell activations, the probability of several rounds of replication grows slowly with the number of activations, see Fig 4b. We therefore expect our qualitative model prediction, of a few rounds of viral replication only before extinction in patients on effective therapy, to be robust to model extensions including clonal expansion.

The stochastic analogue of our simple model of viral replication reveals that there may be a few rounds of viral replication, before lineage extinction. This model prediction is consistent with the observation of recent viral evolution in a genotypic study of episomal HIV cDNA collected prior to viral rebound [23]. However, these few rounds of viral replication leave little opportunity for drug resistance mutants to arise, since single nucleotide substitutions that can lead to drug resistance occur with *O*(10^−5^) probability per round of replication [52, 53], which is also consistent with observations [17, 18].

### The rate of latent reservoir re-seeding is negligible in patients on treatment

#### Model extension 2: Two latent cell compartments

We can also use an extension of the model (4) to determine the extent to which the latent reservoir is re-seeded during treatment. Let *L*_*o*_ represent the pre-existing latent population, and *L*_*n*_ represent new latently infected cells, i.e., the re-seeded population, after time *t* = 0. The ODE model (4) is extended to become

$$\begin{array}{ccc} \frac{dL_{o}}{dt} & = & -\eta_{1}L_{o}\\ \frac{dL_{n}}{dt} & = & -\eta_{1}L_{n}+fR\delta I\\ \frac{dI}{dt} & = & a\left(L_{o}+L_{n}\right)-\delta \left[1-(1-f)R\right]I. \end{array}$$ (14)

The eigenvalues of the system Eq (14) are

$$\begin{array}{ccc} \lambda_{1} & = & -\eta_{1}\\ \lambda_{\pm } & = & \frac{1}{2}\left(-\eta_{1}-\delta \left(1-(1-f)R\right)\pm \sqrt{(\mu_{1}-\delta {\left(1-(1-f)R)\right)}^{2}+4a\delta fR}\right). \end{array}$$

Note that *λ*_±_ remain as before, as in Eq (8), as expected. The associated eigenvectors are

$$\begin{array}{ll} {\vec{v}}_{1} & ={\left(\begin{array}{ccc} -1 & 1 & 0 \end{array}\right)}^{T}\\ {\vec{v}}_{\pm } & =\left(\begin{array}{c} 0\\ 2\delta fR\\ \eta_{1}-\delta (1-(1-f)R)\pm \sqrt{{\left(\eta_{1}-\delta (1-(1-f)R)\right)}^{2}+4a\delta fR} \end{array}\right) \end{array}$$

We previously defined *η*_2_ = −*λ*_+_. Now *λ*_1_ = −*η*_1_ and *λ*_+_ = −*η*_2_ are of the same order, and time scale separation is no longer clear. Further, the separate populations *L*_*o*_ and *L*_*n*_ decay at different rates, so we cannot set an initial condition along the slowest manifold, as was previously done. We therefore take the initial conditions *L*_*o*_(0) = *L*_0_, *L*_*n*_(0) = 0, and *I*_0_ as in eq. (C) (see S1 Text).

#### Rate of latent reservoir re-seeding is negligible

**Fig 5:**
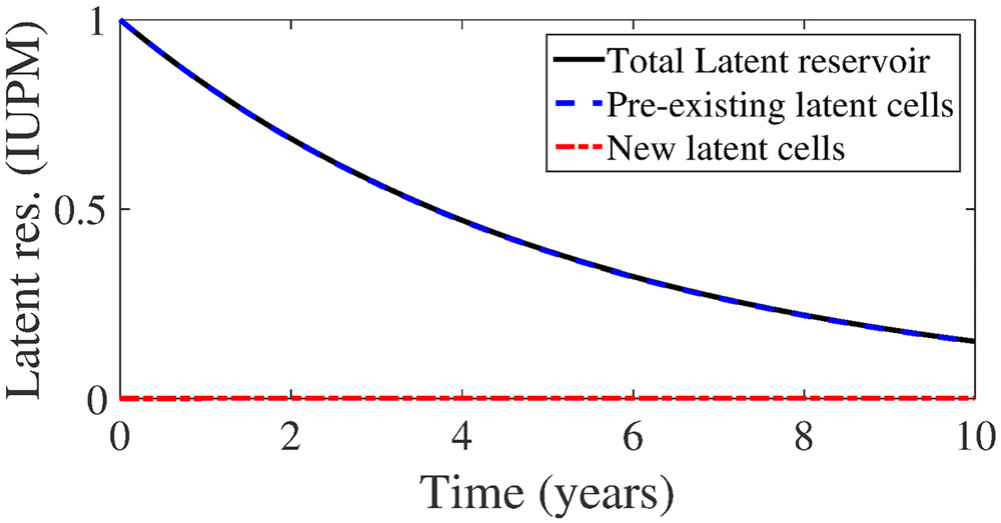
Total latent reservoir size (black, solid line) with pre-existing portion (blue, dashed lines) and new latent cell infections (red, dash-dotted line) for latent cell fraction *f* = 10^−4^ and drug efficacy *ε* = 0.6 (reproductive ratio *R* = 0.92), assuming *δ* = 1 day^−1^ and *t*_1/2_ = 44 months.

In the S1 Text, we show that our model predicts noticeable latent reservoir replenishment in patients on treatment only if *f* quite large, *O*(10^−1^). Fig 5 shows latent reservoir dynamics for the largest realistic fraction *f* = 10^−4^ and drug efficacy *ε* = 0.9, corresponding to a reproductive ratio *R* = 0.23. After 10 years simulation time, the contribution of new latent cells to the total number of latent cells *L*_*n*_/*L* is ≈ .00057%. If the total latent reservoir size is 10^5^ cells, the number of new latently infected cells in the reservoir, on average, is .57, i.e., less than 1.

Even if we assume poor drug efficacy, say *ε* = 0.6, which implies a reproductive ratio *R* = 0.92 and gives the initial residual plasma viral load of 38 copies/mL, the contribution remains negligible. 10-year dynamics are not shown but are almost identical to Fig 5. The contribution of new latently infected cells at the simulation end time *L*_*n*_/*L* is ≈ .022%. If the total latent reservoir size is 10^5^ cells, the number of new latently infected cells in the reservoir, on average, is approximately 22 cells only.

## Discussion

We have presented a simple HIV viral dynamics model, extended from the standard model [29], that recapitulates the following features of HIV infection in patients on suppressive therapy:

- Intensification of drug therapy has no appreciable impact on CD4^+^ counts or viral load on therapy [15, 16]: for reasonable choices of *ε* > 0.99, our model predicts that the contribution of ongoing viral replication is so low, < 2–3%, that decreasing that contribution via therapy intensification (increasing *ε*) should have no discernible impact.
- A genotypic study of episomal HIV cDNA collected prior to viral rebound showed evidence of recent evolution [23]: Our model predicts that a few rounds of viral replication may follow the activation of a latently infected cell (c.f. Fig 4), before the lineage created dies out, which is consistent with this observation.
- During suppressive ART, plasma virus shows little or no development of drug resistance mutations [17, 18] and no evidence of evolution [21]: Following the activation of a latently infected cell, a few rounds of viral replication may follow, but ultimately the viral lineage created goes extinct, as illustrated in Fig 3. Therefore we predict that it is unlikely to observe any change over time from virus archived in the latent reservoir, with the exception of a mutant that confers resistance to therapy. However, the probability of more than a few rounds of viral replication is very low (c.f. Fig 4a), even given our estimate of 174 activations per day (c.f. Fig 4b). Therefore emergent drug resistance, which for a single drug occurs with probability approximately 10^−5^ per round of replication per drug [52, 53]—recall that patients are typically on a combination of three or more drugs [27]—is predicted to be unlikely.

Our primary assumption is that latent cell activation drives viral dynamics on therapy. This assumption is supported by the observation that clonal sequences of plasma virus indicate a close relationship with virus archived in the latent reservoir [19, 20], and is an increasingly well-accepted hypothesis [26, 34, 51, 54].

An important aspect of our analysis is that our results rely primarily on the with (i.e. change in to within) in-host basic reproductive ratio of HIV in patients on effective therapy, *R*. In particular, since the fraction of new infections that result in latency, *f*, is very small [34], the fraction of residual viremia attributable to viral replication in patients on suppressive therapy is approximately *R*, Eq (12). Further, the probability distribution on the number of rounds of replication achievable after the activation of a latently infected cell, before the lineage dies out, is a function of R only. We made a reasonable choice of *R* but no clear estimate exists for patients on suppressive therapy. Our model predicts that estimation of the reproductive ratio of a patient on therapy, rather than individual parameters that make up the ratio (e.g. viral production rate *p*, drug efficacy *ε*) would allow us to effectively characterize ongoing replication in patients on therapy, analyzing for example the probability of emergent drug resistance across different individuals.

The implication of our modeling on the low probability of emergent drug resistance re-enforces results from Ribeiro et al. (2000) [55]. There the authors argued that, since the proportion of infected cells produced over time in patients on ART is very small relative to the number of infected cells in patients pre-therapy, for drug resistant variants to emerge, they most likely already exist in the infected cell population at initiation of therapy. To this argument we add the fact that the proportion of infected cells in patients on therapy that have resulted from any viral replication is approximately *R*, the viral reproductive ratio in patients when on therapy, further reducing the probability of drug resistance emerging from ongoing viral replication.

The assumption of high drug efficacy implies that patients are adherent to therapy, which may not always be the case [56]. Patients who are not adherent, or patients who have developed some resistance to therapy, may have low drug efficacy. In that case we would expect a high reproductive ratio *R* near 1, and therefore a high proportion (approximately *R*) of residual viremia to be associated with ongoing viral replication. We used *ε* = 0.6 as an illustrative example of this case, with *R* = 0.92 and therefore 92% of residual viremia due to ongoing viral replication (see Fig 2c). Although a latent cell activation would be followed, in this case, by more rounds of viral replication than for higher drug efficacy, ultimately the lineage would still die out (see Fig 3b and 3c). More rounds of viral replication implies more chances for a drug resistant variant to emerge, but the probability is still small; there are too few rounds of replication to be assured of the right mutation (see Fig 4). It is important to note however, that *ε* = 0.6 > *ε*_*c*_, the critical drug efficacy below which therapy is not suppressive. Our modeling predictions are contingent on *R* < 1. They are not valid, for example, for cases where adherence to therapy in a patient is such that average drug efficacy dips below this critical value *ε*_*c*_, which gives *R* > 1.

Our model suffers from a number of other limitations. Importantly, we model dynamics of latent cell activation very simply; we assume no clonal expansion, which may occur since latently infected cells are mainly memory cells [9, 51, 57–61], and we assume that an activated latently infected cell is the same as a productively infected cell, which may not be the case. We also assume new latently infected cells decay at the same average rate as pre-existing latent populations. In these pre-existing latent populations, activation by common cognate antigens likely already occurred, yielding a slow activation rate; new latently infected cells may still be specific to common antigens and hence have a more rapid activation rate. It is also a one-compartment model, that is, we do not model dynamics in different tissues individually, in particular lymphatic tissue where drug concentrations may be lower than in blood [25], and where residual replication may occur in productively infected CD4^+^ T cells without being reflected in plasma viremia [24, 25]. Viral and cell transport between tissues may play an important role in promoting HIV infection in patients on therapy [3, 24].

In spite of these limitations, we have shown that our models, with relatively few parameters, recapitulate HIV viral dynamics observed in patients on suppressive therapy. We used a variant of the model to predict that viral replication cannot replenish the reservoir in a patient on therapy. Current strategies for HIV functional cure target the latent reservoir, with reservoir eradication as the goal. Our prediction implies that these reservoir eradication strategies will not be obstructed by latent reservoir replenishment in HIV^+^ patients on effective therapy.

## Supporting Information

S1 Text Supporting information. Supporting mathematical analyses, tables, and figures.
(PDF)
