# Supplementary material for: Residual Viremia in Treated HIV+ Individuals
Source: PLoS Comput Biol. 2016 Jan 6;12(1):e1004677. doi: 10.1371/journal.pcbi.1004677 (PMC4703306; doi:10.1371/journal.pcbi.1004677)
Supplement: S1 Text — Supporting mathematical analyses, tables, and figures. (PDF) [file pcbi.1004677.s001.pdf]

Residual viremia in treated HIV+ individuals
Supporting Information

A Derivation of the reproductive ratio R and the quantity R_L

The reproductive ratios R gives the average number of new cell infections caused by a single infected cell in a completely susceptible population, assuming the standard viral dynamics model [1, 2]. To derive R, given by eq. (2) in the main text, we employ the next generation matrix method [3].

The quantity R_L describes how R is modified assuming the latent reservoir dynamics in eq. (1), i.e., how latent reservoir dynamics affect the average number of new productive cell infections caused by a single productively infected cell. The derivation of R_L is given in Sec. A.2. Note that the quantity R_L is not the reproductive ratio for the standard model extended to incorporate latent reservoir dynamics, eq. (1) in the main text. That reproductive ratio has a more complex interpretation since both latently and productively infected cell represent infectious classes.

A.1 Derivation of R

The basic reproductive ratio R is a well-known quantity [4]. We re-derive it here for the reader's convenience.

The basic reproductive ratio R, given by eq. (2) in the main text, is derived from the standard viral dynamics model [1, 2]

$$\begin{aligned}\frac{dI}{dt} &= (1-\varepsilon)\beta T V - \delta I \\ \frac{dV}{dt} &= pI - (c+\beta T)V.\end{aligned}$$

with the target cells T held constant, using the the next generation method, in which the equations are written in vector form,

$$\frac{d\vec{x}}{dt} = \mathscr{F}(\vec{x}) - \mathscr{V}(\vec{x}),$$

$\vec{x} = \begin{pmatrix} I & V \end{pmatrix}^T$ [3]. The next generation method then gives the reproductive ratio as the spectral radius (maximum eigenvalue) of the matrix $F\tilde{V}^{-1}$, where $F = \frac{d\mathscr{F}}{d\vec{x}}$ is the matrix that captures the rate of appearance of new infections in the compartment I, productively infected cells, and $\tilde{V} = \frac{d\mathscr{V}}{d\vec{x}}$ is the matrix that gives the rate of transfer of individuals between compartments I and V by all other means, and is expressed as the rate of transfer of individuals out of a compartment minus their rate of transfer into the compartment. In this case,

$$F = \begin{pmatrix} 0 & (1-\varepsilon)\beta T \\ 0 & 0 \end{pmatrix}, \tilde{V} = \begin{pmatrix} \delta & 0 \\ -p & c+\beta T \end{pmatrix}, \text{ and } \tilde{V}^{-1} = \begin{pmatrix} 1/\delta & 0 \\ p/(\delta(c+\beta T)) & 1/(c+\beta T) \end{pmatrix}.$$

Then the spectral radius of $F\tilde{V}^{-1}$, and therefore the reproductive ratio, is

$$R = \frac{(1-\varepsilon)\beta T p}{(c+\beta T)\delta},$$

as in eq. (2).

Alternatively, if we assume the concentration of target cells is constant and apply the quasi-steady approximation $V = pI/(c+\beta T)$, our viral dynamics model is reduced to the single ODE,

$$\dot{I} = \frac{(1-\varepsilon)\beta T p}{c+\beta T} I - \delta I.$$

Then using the next-generation matrix method, $F = \frac{(1-\varepsilon)\beta T p}{c+\beta T}$, $\tilde{V} = \delta$, and again $R = F\tilde{V}^{-1} = \frac{(1-\varepsilon)\beta T p}{(c+\beta T)\delta}$.

A.2 R_L: Average number of new cell infections altered by latent reservoir dynamics

The reproductive ratio R computed above is defined as the average number of infected cells I in the second generation, starting from a single infected cell I. The introduction of latent reservoir dynamics in the viral dynamics model, eq. (4)

$$\begin{aligned} \frac{dL}{dt} &= fR\delta I + (\rho - a - \mu)L \\ \frac{dI}{dt} &= aL - \delta\left[1-(1-f)R\right]I, \end{aligned}$$

will alter this average. We define this new average number of productively infected cells I produced by a single I to be R_L.

To calculate R_L, consider, for a moment, the latent cell dynamics in isolation. New latently infected cells are born at rate ρ and clear at rate $a+\mu$. The latent reservoir reproductive ratio is therefore $\rho/(a+\mu)$, which we know to be less than 1, since the latent reservoir is decaying [5]. A single latently infected cell will produce, on average, $\rho/(a+\mu)$ new latently infected cells. The new latently infected cells will, in turn, produce an average of $\rho/(a+\mu)$ new latently infected cells. Proceeding in this manner we find that the total number of latently infected cells produced by a single latently infected cell is given by the geometric series

$$\sum_{j=0}^{\infty}\left(\frac{\rho}{a+\mu}\right)^j = \frac{1}{1-\left(\frac{\rho}{a+\mu}\right)}.$$

But latently infected cells are not isolated; each can activate at rate a or die at rate μ. We're interested in the number of new productively infected cells I that derive from latently infected cells L, which occurs per L with probability $a/(a+\mu)$. Thus, the total number of new productively infected cells that derive from a single latently infected cell is

$$\left(\frac{1}{1-\left(\frac{\rho}{a+\mu}\right)}\right)\left(\frac{a}{a+\mu}\right) = \frac{a}{a+\mu-\rho}.$$

Note that $a+\mu-\rho>0$ since the latent reservoir is decaying.

Finally we compute R_L, the average number of secondary productively infected cells caused by a single productively infected cell. On average, a productively infected cell will produce $(1-f)R$ productively infected cells and fR latently infected cells. But a latently infected cell, on average, produces $a/(a+\mu-\rho)$ productively infected cells. Therefore,

$$R_L = (1-f)R + fR\left(\frac{a}{a+\mu-\rho}\right).$$

After some simplification, we find that

$$R_L = R\left(1-f\left(1-\frac{a}{a+\mu-\rho}\right)\right).$$

B Model (4): analysis and parameter derivation

B.1 Analytic solution of model (4)

The system of equations (4), assuming a constant number of target cells T so that the reproductive ratio R is constant, is a 2x2 linear system which has the general solution

$$\begin{pmatrix} L \\ I \end{pmatrix} = c_1\vec{v}_+ e^{\lambda_+ t} + c_2\vec{v}_- e^{\lambda_- t}$$

where $\lambda_\pm$ are eigenvalues with associated eigenvectors $\vec{v}_\pm$,

$$\lambda_\pm = \frac{1}{2}\left(-\eta_1-\delta\,(1-(1-f)R) \pm \sqrt{(\eta_1-\delta(1-(1-f)R))^2+4a\delta fR}\right) \quad \text{(A)}$$

$$\vec{v}_\pm = \begin{pmatrix} -\eta_1+\delta\,(1-(1-f)R) \pm \sqrt{(\eta_1-\delta(1-(1-f)R))^2+4a\delta fR} \\ 2a \end{pmatrix} \quad \text{(B)}$$

(eigenvectors are not normalized). Since all parameters are positive, and $0<f<1$ and $0<R<1$, one can show that both eigenvalues $\lambda_\pm$ are negative:

$$\lambda_- = -\left(\eta_1+\delta\,(1-(1-f)R)+\sqrt{(\eta_1-\delta(1-(1-f)R))^2+4a\delta fR}\right)/2<0$$

since all quantities inside the parenthesis are positive. The sign of λ_+ is less clear, but still negative: $\lambda_+<0 \Rightarrow$

$$\begin{aligned}
\frac{1}{2}\left(-\eta_1-\delta\,(1-(1-f)R)+\sqrt{(\eta_1-\delta(1-(1-f)R))^2+4a\delta fR}\right) &< 0 \\
\sqrt{(\eta_1-\delta(1-(1-f)R))^2+4a\delta fR} &< \eta_1+\delta\,(1-(1-f)R) \\
(\eta_1-\delta(1-(1-f)R))^2+4a\delta fR &< (\eta_1+\delta\,(1-(1-f)R))^2.
\end{aligned}$$

The last step is true since both sides of the inequality are positive. This last expression simplifies to the inequality

$$\begin{aligned} afR &< \eta_1(1-(1-f)R) \\ \Rightarrow 0 &< \eta_1 - R(\eta_1(1-f)-af). \end{aligned}$$

But $0 \leq R \leq 1$, so $\eta_1 - R(\eta_1(1-f)-af) \geq \eta_1 - (\eta_1(1-f)-af) = (\eta_1+a)f > 0$, since η_1, a, and f are positive. Therefore, $afR < \eta_1(1-(1-f)R)$ and the eigenvalue λ_+ is indeed negative.

The larger eigenvalue λ_+ is closer to zero, and $\vec{v}_+$ is therefore the slow manifold of the fixed point $(L,I) = (0,0)$. We will focus on dynamics along $\vec{v}_+$.

B.2 Quasi-equilibrium initial conditions

We are interested in exploring viral dynamics in patients on long-term treatment. Let $t = 0$ represent an arbitrary time after the initiation of treatment, after transient viral load dynamics have passed, and the viral load is below the detection level of clinical assays. The subsequent dynamics correspond to dynamics along the slow manifold described by the eigenvalue λ_+ and corresponding eigenvector $\vec{v}_+$. Therefore, to investigate viral dynamics in patients on long-term treatment, our initial conditions should lie along this slow manifold, with I_0 and L_0 in quasi-equilibrium. To this end, if we choose the number of latently infected cells to be L_0 at $t = 0$, we need to set the number of productively infected cells at $t = 0$, I_0, so that it is on the slow manifold, i.e., so that $L_0/I_0 = \vec{v}_+^{(1)}/\vec{v}_+^{(2)}$, where $\vec{v}_+ = \begin{pmatrix} \vec{v}_+^{(1)} & \vec{v}_+^{(2)} \end{pmatrix}$, that is,

$$I_0 = \frac{2aL_0}{-\eta_1 + \delta(1-(1-f)R) + \sqrt{(\eta_1 - \delta(1-(1-f)R))^2 + 4a\delta fR}}. \tag{C}$$

B.3 Latent reservoir dynamics: activation rate a and decay rate η_1

Other than the latent reservoir net half-life, $t^L_{1/2}$, model parameters relating to latent reservoir dynamics, η_1 and a, remain unclear. First we consider η_1. Let $\eta_2 = \ln(2)/t^L_{1/2}$ be the net decay rate of the latent reservoir when replenishment by de novo infection is considered, so that $L(t) \sim e^{-\eta_2 t}$. In our model, $-\eta_2$ must correspond to the slowest decay, i.e., the largest eigenvalue, λ_+ (eq. (A)), i.e.,

$$\eta_2 = -\lambda_+ = -\frac{1}{2}\left(-\eta_1 - \delta(1-(1-f)R) + \sqrt{(\eta_1 - \delta(1-(1-f)R))^2 + 4a\delta fR}\right). \tag{D}$$

Using this equation to determine η_1, the latent reservoir decay rate in the absence of replenishment, we find

$$\eta_1 = \eta_2 - \frac{a\delta fR}{\eta_2 - \delta(1-(1-f)R)}. \tag{E}$$

Since $\eta_2 = \ln(2)/t^L_{1/2}$, and there are established estimates for both $t^L_{1/2}$ and the infected cell death rate δ, we obtain an equation for the parameter η_1 as a function of f, a, and R. If the fraction $f = 0$, $\eta_2 = \eta_1$, which is expected since $\eta_1 = -(\rho - a - \mu)$ is the latent reservoir decay rate in absence of replenishment by de novo infection.

To determine the activation rate a we employ the quasi-steady assumption $I = cV/p$, $I_0 = cV_0/p$, neglecting βT since $c \gg \beta T$. Substituting this and (E) in to eq. (C) we find

$$a = (\delta(1-(1-f)R) - \eta_2)\frac{cV_0}{pL_0}, \tag{F}$$

C Infected cell lineage distribution if $R < 1$

If the reproductive ratio $R < 1$, the lineage of infected cells generated from the activation of a single latently infected cell goes extinct as $t \to \infty$. However, when considering the possibility of a sequence of viral mutations that may result in a reproductive ratio $R > 1$ for a mutant, for example by conferring resistance to ongoing therapy, the number of generations before the lineage goes extinct becomes important. The number of generations represents the number of "chances" for the drug resistance mutations to take place.

To compute the number of generations we employ the branching process analogue of the differential equations model assuming a constant number of target cells T,

$$\begin{aligned} \dot{I} &= \beta T V - \delta I \\ \dot{V} &= pI - (c + \beta T)V, \end{aligned}$$

which can be expressed as a series of reactions,

$$\begin{aligned} V &\xrightarrow{\beta T} I \\ I &\xrightarrow{\delta} \emptyset \\ I &\xrightarrow{p} I + V \\ V &\xrightarrow{c} \emptyset. \end{aligned}$$

The branching process is subcritical since $R = (1-\varepsilon)p\beta T/\delta(c+kT) < 1$ (eq. (2), so the lineage will go extinct as $t \to \infty$. Our aim is to compute the probability distribution of the number of generations before extinction in this subcritical branching processes [6].

To compute this probability distribution, we require the offspring distribution, that is, the distribution of the number of infected cells in the first generation following the activation of a latently infected cell into a productively infected cell. Assuming n viral progeny from a single productively infected cell, the number of new infected cells (offspring) j is given by the binomial distribution probability $q_{j,n} = \left(\begin{array}{c} n \\ j \end{array}\right)\left(\frac{\beta T}{c+\beta T}\right)^j\left(\frac{c}{c+\beta T}\right)^{n-j}$: j virions infect cells, $n-j$ clear. So the probability generating function [7] for the number of infected offspring, assuming n viral progeny for each infected cell, is

$$\tilde{h}(x) = \sum_{j=0}^{n} q_{j,n} x^j.$$

The number of viral progeny before infected cell death is given by a geometric distribution with probability $r_n = \left(\frac{p}{p+\delta}\right)^n\left(\frac{\delta}{p+\delta}\right)$. The probability generating function for the number of infected

offspring from a single infected cell is therefore

$$
\begin{aligned}
h(x) &= \sum_{n=0}^{\infty} r_n \sum_{j=0}^{n} q_{j,n} x^j \\
&= \left(\frac{\delta}{p+\delta}\right) \sum_{n=0}^{\infty} \left(\frac{p}{p+\delta}\right)^n \sum_{j=0}^{n} \binom{n}{j} \left(\frac{\beta T}{c+\beta T}\right)^j \left(\frac{c}{c+\beta T}\right)^{n-j} x^j \\
&= \left(\frac{\delta}{p+\delta}\right) \sum_{n=0}^{\infty} \left[\left(\frac{p}{p+\delta}\right)\left(\frac{c}{c+\beta T}\right)\right]^n \sum_{j=0}^{n} \binom{n}{j} \left(\frac{\beta T}{c} x\right)^j \\
&= \left(\frac{\delta}{p+\delta}\right) \sum_{n=0}^{\infty} \left[\left(\frac{p}{p+\delta}\right)\left(\frac{c}{c+\beta T}\right)\left(\frac{\beta T}{c} x + 1\right)\right]^n \\
&= \left(\frac{\delta}{p+\delta}\right) \left(\frac{1}{1-\left(\frac{p}{p+\delta}\right)\left(\frac{c}{c+\beta T}\right)\left(\frac{\beta T}{c} x + 1\right)}\right) \\
&= \frac{1}{1+R(1-x)}, \qquad \text{(G)}
\end{aligned}
$$

summing the series and recalling that $R = p\beta T/\delta(c+kT)$.

To obtain the probability distribution on the number of generations until extinction, we follow [6]. Define α_j as the number of individuals in generation j and let G denote the number of generations to extinction, so that $G = k$ when $\alpha_k \geq 1$ and $\alpha_{k+1} = 0$. The cumulative generation distribution from a single initial case is defined by $f_k = \Pr\{G \leq k | \alpha_0 = 1\}$. Then

$$f_k = h(f_{k-1}), \qquad \text{(H)}$$

$f_{-1} = 0$, where $h(x)$ is the probability generating function for the offspring distribution, eq. (G) [6]. The probability of the lineage surviving to the kth generation, f_k, depends on the probability of surviving to the (k-1)st generation, f_{k-1}, and the distribution on the number of infected offspring from that generation. Then if we start with α_0 cells, from the branching property, the cumulative generation distribution will be $f_k^{\alpha_0}$. Solving the recurrence relation eq. (H) we find

$$f_k = \frac{1-R^{k+1}}{1-R^{k+2}}. \qquad \text{(I)}$$

For example, from the recurrence relation eq. (H), $f_0 = 1/(1+R)$, which we also recover from eq. (I), $f_0 = (1-R)/(1-R^2)$, after noting that $1-R^2 = (1-R)(1+R)$. Finally, define $g_k = \text{Prob}\{G = k\}$, the probability that there are exactly k generations, given α_0 cells in the first generation. Then $g_k = f_k^{\alpha_0} - f_{k-1}^{\alpha_0}$. If we start with a single infected cell, i.e., $\alpha_0 = 1$, we can solve from eq. (I) to find

$$
\begin{aligned}
g_k &= f_k - f_{k-1} \\
&= \frac{1-R^{k+1}}{1-R^{k+2}} - \frac{1-R^k}{1-R^{k+1}} \\
g_k &= \frac{R^k(R-1)^2}{\left(1-R^{k+1}\right)\left(1-R^{k+2}\right)}.
\end{aligned}
$$

Thus, given a basic reproduction number $R < 1$, we can compute the probability that the lineage created by a single infected cell has k generations - k chances to mutate to a drug resistant mutant - before going extinct.

D Model predictions using arbitrary values for f

We investigate the contribution of ongoing viral replication to viral load and latent reservoir replenishment in patients on treatment. Here we use arbitrary values of the latent cell fraction f. Figure 2 shows the total viral load and contributions from viral production by newly infected cells (viral replication) and by activated latent cells. The viral load V is calculated using the quasi-steady assumption $V = pI/(c+\beta T)$. Results shown in Fig. 2 assume that the fraction of infections that lead to latency $f = 10^{-4}$, but the qualitative results shown are not sensitive to f. As shown in Fig. 2, for increasing R, the viral replication contribution to the viral load increases. The fraction of circulating infected cells attributable to viral replication - indicating the viral replication contribution to the viral load - is given in Table A. Note that the approximation we derive, $I_r/I \approx R$, is reasonably good even for unrealistically large $f = 0.1$. Viral production by new cell infections only has a greater contribution than viral production in latent cells for $R > R_c$, ≈ 0.5 (R_c is given by eq. (10)).

Table A: Fraction of circulating virus attributable to newly infected cells I_r/I at equilibrium for different reproductive ratios, drug efficacies ε, and latent fractions f. The associated reproductive ratio is also given. Other parameters are as in Table 1. Note that the critical value of R, R_c (eq. (10)), above which circulating virus is dominantly produced by newly infected cells, is $R_c = 0.50, 0.50, 0.50, 0.56$ for $f = 10^{-6}$, 10^{-4}, 10^{-3}, and 10^{-1}, respectively.

	$\varepsilon = 0.999$ ($R = 0.0023$)	$\varepsilon = 0.99$ ($R = 0.023$)	$\varepsilon = 0.9$ ($R = 0.23$)
$f = 10^{-6}$	0.0023	0.0230	0.2301
$f = 10^{-4}$	0.0023	0.0230	0.2301
$f = 10^{-3}$	0.0023	0.0230	0.2299
$f = 10^{-1}$	0.0021	0.0207	0.2071

In Fig. A we show the latent reservoir size, with pre-existing portion and the portion coming from reservoir replenishment via viral replication, i.e. the fraction of new cell infections becoming latently infected cells, for $f = 0.1$ (which is unrealistically high) and decreasing drug efficacy ε, i.e., increasing reproductive ratio R. As the reproductive ratio increases so does the latent reservoir replenishment. These quantitative results are quite sensitive to f : for smaller f, the new latent cells curve remains very near 0, even for $R > R_c$ (not shown). However only for unrealistically high values of f, i.e. $f = 0.1$, do we note any discernible contribution of ongoing viral replication to the latent reservoir, as in Fig. Ac.

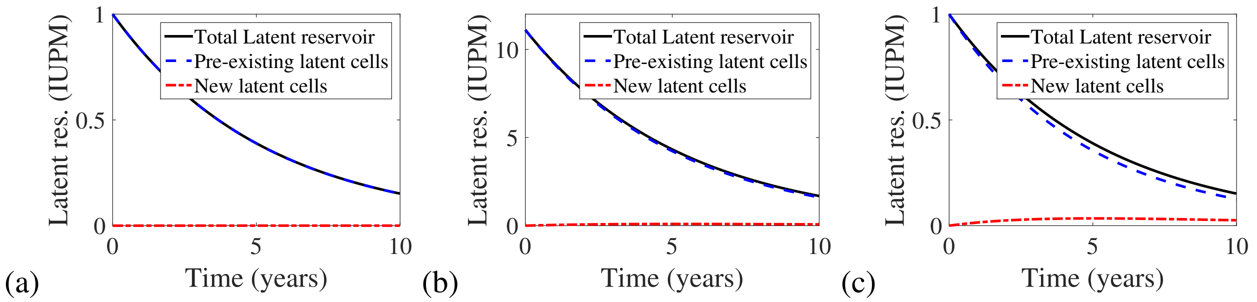

Figure A: Total latent reservoir size (black, solid line) with pre-existing portion (blue, dashed lines) and new latent cell infections (red, dash-dotted line) for latent cell fraction $f = 0.1$ and drug efficacy (reproductive ratio) (a) $\varepsilon = 0.999$ ($R = 0.0023$), (b) $\varepsilon = 0.99$ ($R = 0.023$), and (c) $\varepsilon = 0.9$ ($R = 0.0023$). Other parameters set to $\delta = 1$ day^{-1} and $t_{1/2}$ =44 months.

The model predicts that it is theoretically possible, in patients on treatment, for a substantial portion of the viral load to be associated with viral replication, and for the contribution of new cell infections to the latent reservoir to be non-negligible. But in making these theoretical predictions we used an arbitrary range in latent cell fraction f and reproductive ratio R. The upper ranges used, i.e., $f = 0.1$, are likely not very realistic. Observations, for example in Joos et al. [8], suggest that there is very little evolution in the viral genome in patients on treatment, implying that theoretical scenarios with large contributions from viral replication - e.g. Fig. A with large f - are not likely. In Sec. 3 we discuss realistic values for these parameters, and show associated predictions in the main text.

E Parameter choice: baseline drug efficacy ε

For our simulation results, we employ a latent cell activation rate a that is computed using eq. (6) assuming a drug efficacy $\varepsilon = 0.99$. With the recent exception of raltegravir [9], an integrase inhibitor, drug efficacy is poorly characterized and our choice of $\varepsilon = 0.99$ is an educated guess. Here we assume alternative baseline drug efficacies $\varepsilon = 0.6,\ 0.7,\ 0.8,\ 0.9,\ 0.999,\ 0.9999$, and 1, and verify that the qualitative results we report in the main text are insensitive to this choice.

Table B shows the activation rates computed from eq. (6),

$$a = (\delta(1-(1-f)(1-\varepsilon)R^*) - \eta_2)\,\frac{cV_0}{pL_0}$$

for $\varepsilon = 0.6,\ 0.7,\ 0.8,\ 0.9,\ 0.999,\ 0.9999$, and 1. The critical drug efficacy ε_c, below which the drug regimen is not suppressive, is $\varepsilon \approx 1 - 1/R^* = 0.57$ (the approximation arises because we use $R = (1-\varepsilon)R^*$, neglecting latent reservoir dynamics in R_L, eq. (3), and in the critical drug efficacy for the full model,

$$\varepsilon_c^L = 1 - \frac{1}{R^*}\left(\frac{a+\mu-\rho}{a+(\mu-\rho)(1-f)}\right).$$

Note that the relationship is linear.

Observe that in Table B, the initial number of latent activations per day aL_0 remains relatively of the same order, or less, than the $aL_0 = 174$ discussed in the main text using baseline $\varepsilon = 0.99$.

Table B: Latent cell activation rates a computed from eq. (6) with different baseline assumption for drug efficacy ε with residual viremia V_0 =3.1 copies/mL and a latent reservoir size of L_0 =1 per 10^6 CD4+ T-cells. Remaining parameters as in Table 1.

Baseline drug efficacy ε	Reproductive ratio R	Activation rate a from eq. (6)	Average # of latent cell activations per day, aL_0
1	0	1.782×10^{-3}	178
0.9999	0.00023	1.781×10^{-3}	178
0.999	0.0023	1.777×10^{-3}	178
0.99	0.023	1.741×10^{-3}	174
0.9	0.23	1.372×10^{-3}	137
0.8	0.46	9.62×10^{-4}	96
0.7	0.69	5.52×10^{-4}	55
0.6	0.92	1.42×10^{-4}	14

Recall from Sec. 5.3 that this rate gives the number of HIV lineages initiated per day, that may go through some few rounds of viral replication before dying out, as illustrated in Fig. 3. These rounds of replication represent chances for mutations including resistance mutations to rise. We found that for realistic values of $R < 1$, the number of rounds of viral replication is small, implying that it is highly improbable that a drug resistant mutant will emerge from a single latent cell activation. However, the probability of having a greater number of rounds of viral replication increases with the number of latent cell activations, but since aL_0 for these different baseline ε values does not dramatically increase, the results of our stochastic model analysis in Sec. 5.3 hold: consistency with observations of recent viral evolution in patients on ART [10], but with little opportunity for drug resistance mutants, which occur with probability $O(10^{-5})$ [11, 12], before the lineages die out.

The baseline assumption on ε, which changes the assumed latent cell activation rate a as shown in Table B, does not change the model prediction eq. (11),

$$\frac{I_r}{I_a + I_r} \approx R,$$

that is, that the proportion of residual viremia associated with viral replication is approximately the reproductive ratio R. That model prediction relies only on the assumption that $f \ll 1$. SI Figures B and C replicate Fig. 2 in the main text, which shows the predicted contribution to total viral load from residual replication and latent cell activation, this time assuming the unrealistic, extreme baseline drug efficacies $\varepsilon = 0.6$ and $\varepsilon = 1$ from Table B. The quantitative results, total viral loads, differ from those in Fig. 2: if we fix a assuming that the baseline drug efficacy of $\varepsilon = 0.6$ gives a viral load of 3.1 copies/mL, improving drug efficacies up to $\varepsilon = 0.9$ and $\varepsilon = 0.99$ decrease total viral load below 3.1 copies/mL. Conversely, if we fix a assuming that the baseline drug efficacy of $\varepsilon = 1$ gives a viral load V_0 of 3.1 copies/mL, diminished drug efficacies of $\varepsilon = 0.9$ and $\varepsilon = 0.99$ must permit viral loads higher than 3.1 copies/mL. However, the qualitative result remains, i.e. that the contribution of residual replication to total viral load is approximately R.

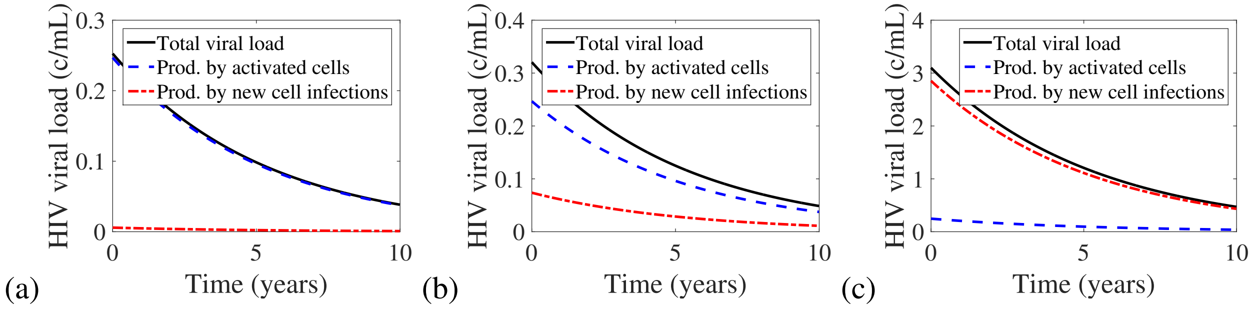

Figure B: Contributions of latent cell activation and residual replication assuming baseline drug efficacy $\varepsilon = 0.6$. Total HIV RNA copies per mL (black, solid line), on a linear scale, with contributions from activated latently infected cells (blue, dashed line) and from newly infected cells (red, dash-dotted lines) for fraction of infections leading to latency $f = 10^{-4}$ and drug efficacy (a) $\varepsilon = 0.99$ ($R = 0.023$, $V_0 \approx 0.25$ copies/mL), (b) $\varepsilon = 0.9$ ($R = 0.23$, $V_0 \approx 0.32$ copies/mL), and (c) $\varepsilon = 0.6$ ($R = 0.92$, $V_0 \approx 3.1$ copies/mL). The activation rate a is chosen so that the initial viral load is $V_0 = 3.1$ copies/mL assuming baseline drug efficacy $\varepsilon = 0.6$, with other parameters as in Table 1.

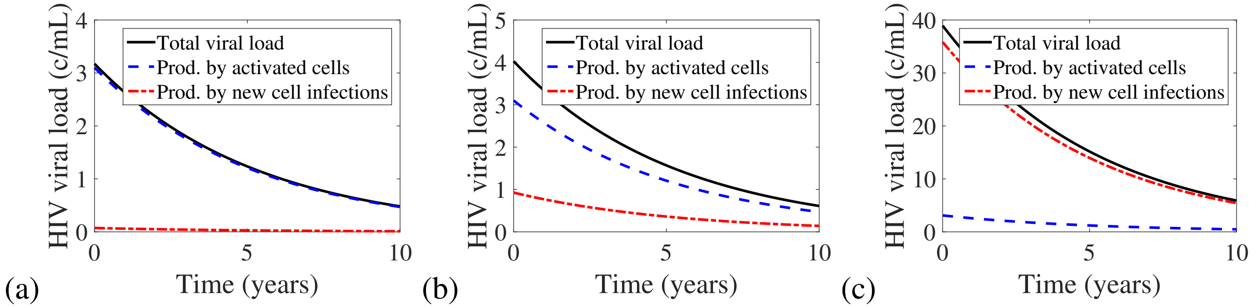

Figure C: Contributions of latent cell activation and residual replication assuming baseline drug efficacy $\varepsilon = 1$. Total HIV RNA copies per mL (black, solid line), on a linear scale, with contributions from activated latently infected cells (blue, dashed line) and from newly infected cells (red, dash-dotted lines) for fraction of infections leading to latency $f = 10^{-4}$ and drug efficacy (a) $\varepsilon = 0.99$ ($R = 0.023$, $V_0 \approx 3.2$ copies/mL), (b) $\varepsilon = 0.9$ ($R = 0.23$, $V_0 \approx 4$ copies/mL), and (c) $\varepsilon = 0.6$ ($R = 0.92$, $V_0 \approx 40$ copies/mL). The activation rate a is chosen so that the initial viral load is $V_0 = 3.1$ copies/mL assuming baseline drug efficacy $\varepsilon = 1$, with other parameters as in Table 1.

F Supporting figures

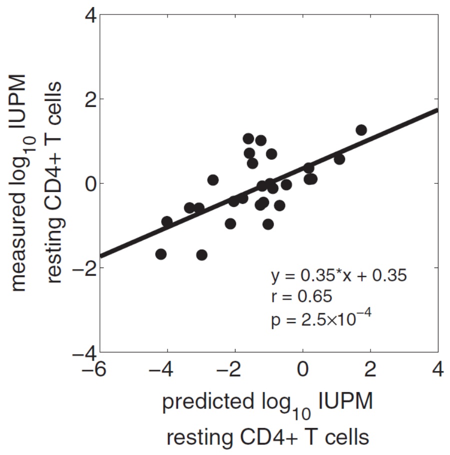

Figure D: Reproduced from Archin et al. (PNAS 2012) [13], Fig. 2. Caption: Correlation between model prediction and the measured frequency of latently infected cells. Predicted infectious units per million resting CD4+ cells (IUPM) was computed as $[L(t_F)/f\beta T(t_F)] \times 10^6$, i.e., the fraction of CD4+ cells at the time of leukopheresis, t_F, that are predicted to be latently infected in 1 million resting CD4+ T cells, and is expressed in arbitrary units of $10^{14} f\beta$.

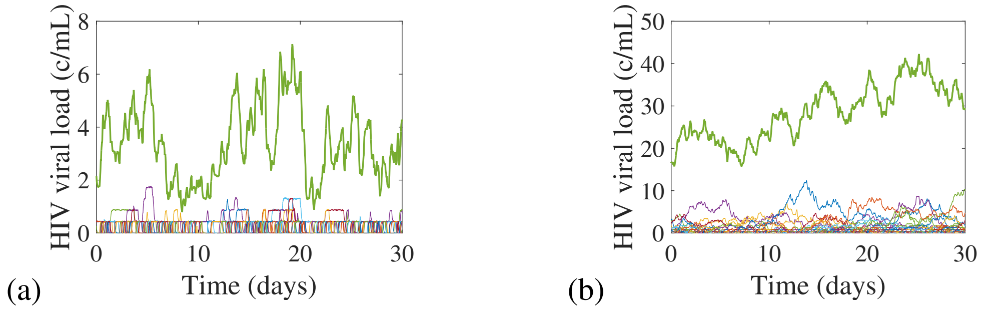

Figure E: Stochastic simulation realization [14] of viral load dynamics for (a) drug efficacy $\varepsilon = 0.9$, associated with $R = 0.23$ and mean viral load $V_0 \approx 4$ copies/mL, and (b) $\varepsilon = 0.6$, associated with $R = 0.92$ and mean viral load $V_0 \approx 38$ copies/mL. The green, thick line gives total viral load, the sum of the viral output from rounds of replication resulting from latent cell activations, shown at the bottom of each figure. Note that in each case, the latent cell activations occur at the same time; the larger mean viral load in (b) is caused by the larger number of rounds of replication following each latent cell activation for $R = 0.92$. The latent reservoir decay rate $\eta_2 = \log(2)/44$ months and aL_0 is chosen so that the initial viral load is $V_0 = 3.1$ copies/mL assuming baseline drug efficacy $\varepsilon = 0.99$, infection cell death rate $\delta = 1$ day^{-1}, and latency fraction $f = 10^{-4}$.

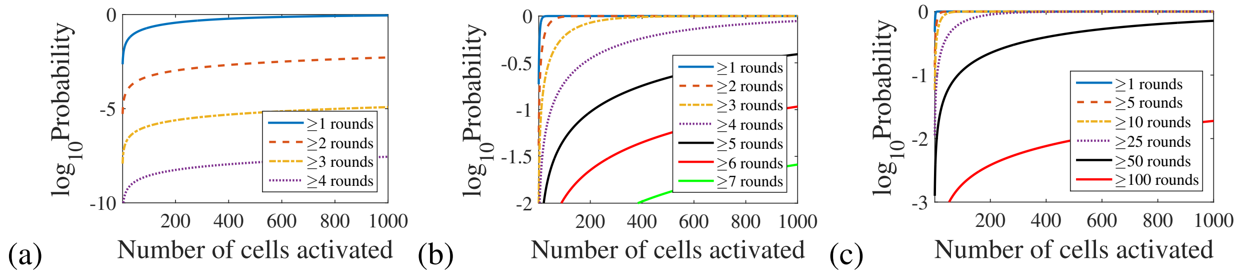

Figure F: Probability distribution on number of viral replication following activation of latently infected cells. (a) The probability on the maximum number of rounds of viral replication achieved as a function of the number of latent cell activations, using (a) drug efficacy $\varepsilon = 0.999$, associated with basic reproductive ratio $R = 0.0023$ and initial viral load $V_0 \approx 3$ copies/mL, (b) $\varepsilon = 0.9$, associated with $R = 0.23$ and $V_0 \approx 4$ copies/mL, and (c) $\varepsilon = 0.6$, associated with $R = 0.92$ and $V_0 \approx 38$ copies/mL. The latent reservoir decay rate $\eta_2 = \log(2)/44$ months and aL_0 is chosen so that the initial viral load is $V_0 = 3.1$ copies/mL assuming baseline drug efficacy $\varepsilon = 0.99$, infection cell death rate $\delta = 1$ day^{-1}, and latency fraction $f = 10^{-4}$. Note that these are discrete distribution functions, with the dots in (a) indicating probability of cells achieving k generations; the lines are included for clarity and have no meaning.

References

[1] Perelson AS, Kirschner DE, De Boer RJ. Dynamics of HIV-infection of CD4+ T-cells. Math Biosci. 1993;114(1):81–125.

[2] Perelson AS, Neumann AU, Markowitz M, Leonard JM, Ho DD. HIV-1 dynamics in vivo: virion clearance rate, infected cell life-span, and viral generation time. Science. 1996;271(5255):1582–1586.

[3] Diekmann O, Heesterbeek JAP. Mathematical Epidemiology of Infectious Diseases: Model Building, Analysis and Interpretation. Wiley; 2000.

[4] Nowak M, May RM. Virus Dynamics: Mathematical Principles of Immunology and Virology. Oxford University Press; 2001.

[5] Siliciano JD, Kajdas J, Finzi D, Quinn TC, Chadwick K, Margolick JB, et al. Long-term follow-up studies confirm the stability of the latent reservoir for HIV-1 in resting CD4+ T cells. Nat Med. 2003;9(6):727–728.

[6] Farrington CP, Grant AD. The distribution of time to extinction in subcritical branching processes: applications to outbreaks of infectious disease. J Appl Probab. 1999;36:771–779.

[7] Karlin S, Taylor HM. A First Course in Stochastic Processes, Second Edition. Academic Press; 1975.

[8] Joos B, Fischer M, Kuster H, Pillai SK, Wong JK, Boeni J, et al. HIV rebounds from latently infected cells, rather than from continuing low-level replication. Proc Natl Acad Sci USA. 2008;105(43):16725–16730.

[9] Andrade A, Guedj J, Rosenkranz SL, Lu D, Mellors J, Kuritzkes DR, et al. Early HIV RNA decay during raltegravir-containing regimes exhibits two distinct subphases (1a and 1b). AIDS. 2015;29:2419–2426.

[10] Llibre JM, Buzón MJ, Massanella M, Esteve A, Dahl V, Puertas MC, et al. Treatment intensification with raltegravir in subjects with sustained HIV-1 viraemia suppression: a randomized 48-week study. Antivir Ther. 2012;17:355–364.

[11] Mansky LM, Temin HM. Lower in vivo mutation rate of human immunodeficiency virus type 1 than that predicted from fidelity of purified reverse transcriptase. J Virol. 1995;69:5087–5094.

[12] Rosenbloom DIS, Hill AL, Rabi SA, Siliciano RF, Nowak MA. Antiretroviral dynamics determines HIV evolution and predicts therapy outcome. Nat Med. 2012;18:1378–1385.

[13] Archin NM, Vaidya NK, Kuruc JD, Liberty AL, Wiegand A, Kearney MF, et al. Immediate antiviral therapy appears to restrict resting CD4+ cell HIV-1 infection without accelerating the decay of latent infection. Proc Natl Acad Sci USA. 2012;109(24):9523–9528.

[14] Gillespie DT. Exact stochastic simulation of coupled chemical reactions. J Phys Chem. 1977;81:2340–2361.
